# Fiber-Type Transistor-Based Chemical and Physical Sensors Using Conjugated Polymers

**DOI:** 10.3390/polym15204062

**Published:** 2023-10-12

**Authors:** Ky Van Nguyen, Donggeun Lee, Youngnan Kim, Wi Hyoung Lee

**Affiliations:** Department of Organic and Nano System Engineering, School of Chemical Engineering, Konkuk University, Seoul 05029, Republic of Korea

**Keywords:** fiber, transistor, conjugated polymer, chemical sensor, sweat sensor, physical sensor

## Abstract

Fiber-type electronics is a crucial field for realizing wearable electronic devices with a wide range of sensing applications. In this paper, we begin by discussing the fabrication of fibers from conjugated polymers. We then explore the utilization of these fibers in the development of field-effect and electrochemical transistors. Finally, we investigate the diverse applications of these fiber-type transistors, encompassing chemical and physical sensors. Our paper aims to offer a comprehensive understanding of the use of conjugated polymers in fiber-type transistor-based sensors.

## 1. Introduction

Transistors are among electronic devices’ most critical components, serving as amplifiers and switches [1]. Silicon-based metal-oxide-semiconductor field-effect transistors (MOSFETs) are extensively employed in driving displays and manufacturing volatile memories such as dynamic random-access memories (DRAM), in which an MOSFET is in conjunction with a capacitor to create a DRAM cell. MOSFETs can also be applied to make non-volatile memory components like NAND flash memory in a floating gate configuration [1]. MOSFETs consist of semiconductor, insulator, and electrode layers, with the semiconductor layer being the key component, typically composed of single-crystalline silicon due to its excellent charge carrier mobility. However, their high manufacturing temperature and cost limitations have spurred research into the development of alternative semiconductors such as amorphous or polycrystalline silicon (poly-Si) [1] and indium gallium zinc oxide (IGZO) [2]. When these materials are used in the channel layer, MOSFETs can be called thin-film transistors (TFT). TFT devices using IGZO have been used in the backplane of flat panel displays [2].

Recently, organic semiconductors have garnered significant interest as the active layer of these transistors due to their various advantages, including flexibility, low cost, room-temperature solution processing, and the ability to control electrical properties through molecular structure manipulation [3,4]. These organic semiconductors combine the electrical properties of traditional semiconductors with the mechanical properties of plastics, making them suitable for flexible electronic devices on unconventional substrates such as fibers and textiles [5]. High-molecular-weight organic semiconductors or conjugated polymers, including poly(3-hexylthiophene) (P3HT), poly(3,4-ethylenedioxythiophene): poly(styrene sulfonate) (PEDOT:PSS), polypyrrole (PPy), and polyaniline, are widely employed in fiber-based electronic devices due to their high flexibility, low density, ease of fabrication, and wide-scale availability [6]. These conjugated polymers can be readily processed into fiber form using methods like electrospinning, electrohydrodynamic (EHD) jet printing, wet spinning, or dip coating. These fibers serve as the building blocks for creating fiber-type transistors employed in various sensing applications.

Fiber-type transistors find application in electronic textiles or fabric systems across multiple applications. They can be seamlessly woven into various fabrics, offering the potential to create electronic devices that are deformable, lightweight, breathable, washable, and comfortable for electronic textiles [7]. Fiber-type sensors can be integrated into fabrics during the manufacturing process, rendering them nearly indistinguishable from regular textiles. Additionally, the cylindrical shape of fiber-type sensors results in a high surface-to-volume ratio, enhancing their selectivity and response time when employed as chemical or biological sensors.

There are several review articles focusing on various aspects of textile-based electronics. For instance, Heo et al. [8] (2018) and Kang et al. [7] (2021) covered applications of fiber-shaped devices in areas like energy harvesting/storage, displays, sensing, and light-emitting devices. Zhang et al. [6] (2022) concentrated on fiber-based transistors in logic gates, memory, and neuromorphic computing. However, a comprehensive review concerning fiber sensors employing conjugated polymer transistors has not yet been published. Therefore, in this review, we present an overview of fiber-type transistor-based sensors that utilize conjugated polymers as their active elements. We begin by focusing on the preparation methods used to produce conjugated polymer fibers. Subsequently, we explore the structure and working principles of fiber-based transistors. Finally, we investigate the various applications of these transistors, particularly in the field of chemical and physical sensing.

## 2. Conjugated Polymer Fibers

### 2.1. Conjugated Polymers

**Figure 1:**
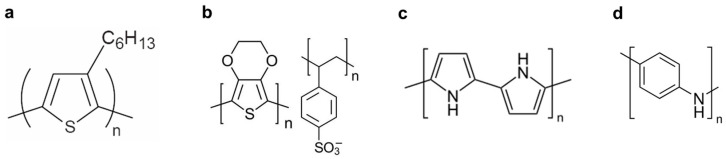
(**a**) Chemical structures of typical conjugated polymers. (**a**) P3HT, (**b**) PEDOT:PSS, (**c**) polypyrrole, and (**d**) polyaniline.

Organic semiconductors consist of a conjugated structure with alternating single and double bonds, allowing for the continuous overlap of p-orbitals. This creates pathways for π-electrons to move. Organic semiconductors can be further divided into small molecular semiconductors and polymeric semiconductors (namely, conjugated polymers) based on the length of the molecular chain. Small molecular semiconductors, such as pentacene, exhibit high field-effect mobility. However, one of the major drawbacks of pentacene is its poor solubility, which prevents its application in solution processing under ambient conditions. The addition of alkyl groups to these materials enhances solubility for solution processing. Conjugated polymers, including poly(3-hexylthiophene) (P3HT), poly(3,4-ethylenedioxythiophene):poly(styrenesulfonate) (PEDOT:PSS), polypyrrole (PPy), and polyaniline, whose chemical structures are shown in Figure 1, are commonly used in fabricating fibers due to advantages such as flexibility, low density, ease of fabrication, and wide-scale availability [6]. 

P3HT is widely used as a solution-processed conjugated polymer for applications such as transistors and solar cells. The chemical structure of P3HT is shown in Figure 1a. On the other hand, PEDOT:PSS, polypyrrole, and polyaniline are primarily used as conductors rather than semiconductors in flexible electrodes. The conductivity of these conjugated polymers increases with dopant concentration [9]. In their undoped state (pure form), they exhibit anisotropic, quasi-one-dimensional electronic structures with a moderate bandgap of 2–3 eV, similar to conventional semiconductors. However, during the doping process, the π bonds change from a non-linear excitation as polarons, solitons, bipolarons, etc., to a metallic state [9]. PEDOT:PSS, with the chemical structure shown in Figure 1b, has gained attention in electronic textiles due to its high conductivity, excellent mechanical flexibility, and long-term thermal stability. PEDOT is a p-type semiconductor and is insoluble in water. Its solubility is improved by adding PSS, a polyelectrolyte, with sulfonic anions in its chains compensating for holes in the PEDOT chains. PEDOT:PSS has been used in textiles through processes like soaking, dipping, and immersing. These applications often utilize textile templates or substrates such as cotton and nylon. Additionally, PEDOT:PSS can be produced in a fiber or filament form using wet spinning and electrospinning methods. For more information about PEDOT:PSS conductive fibers, please refer to a review paper [10,11]. Polypyrrole (Figure 1c) is one of the most extensively studied conductive polymers, thanks to the aqueous solubility of its monomer, low-oxidation potential, and high conductivity [12]. Polypyrrole, with its high-electrical conductivity and environmental stability, has found use in various applications, including rechargeable battery electrodes, electromagnetic shielding materials, and sensors. Polyaniline (Figure 1d) is a well-known conducting polymer applied in various fields, including lightweight battery electrodes, electromagnetic shielding devices, anticorrosion coatings, and chemiresistive sensing [13]. Polyaniline’s electrical conductivity is achieved through doping and protonation, and it can be controlled by factors such as its electrochemical redox state, pH, humidity, temperature, and the type of anions in the solution [10]. For more information on the applications of conjugated polymers, please refer to a review paper [14]. Conjugated polymers can be processed to produce fiber form using various techniques, including electrospinning, electrohydrodynamic (EHD) jet printing, wet spinning, and other coating methods as illustrated in the following section.

### 2.2. Methods for Fabricating Conjugated Polymer Fibers

We focus on fibers consisting only of conjugated polymers and fibers that act as a template onto which a conjugated polymer is coated. Common methods for producing fibers from conjugated polymers include electrospinning, electrohydrodynamic (EHD) jet printing, wet spinning, and other coating methods including vapor polymerization deposition, in situ polymerization, and dip coating. The details of each method will be described as follows.

#### 2.2.1. Electrospinning

**Figure 2:**
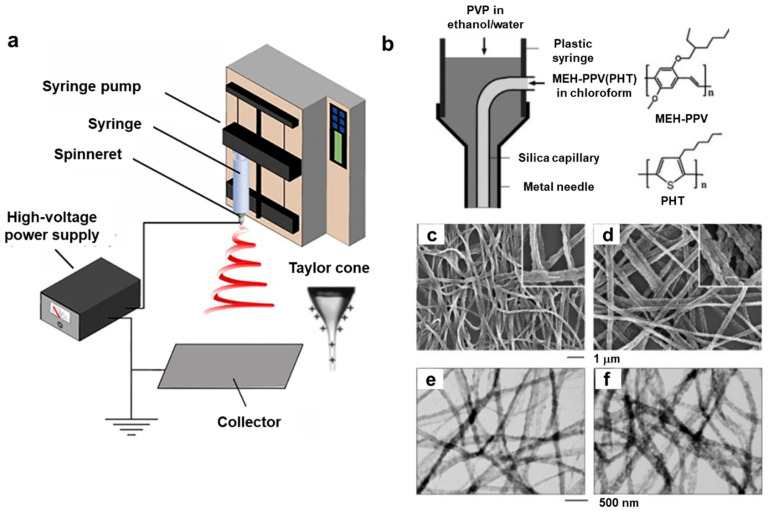
(**a**) Schematic diagram of a typical electrospinning process. Reproduced with permission from Xue et al. [16]. Copyright © 2017, American Chemical Society. (**b**) Schematic illustration of the spinneret with two coaxial capillaries. The molecular structures of MEH-PPV and P3HT are shown on the right. (**c**,**d**) SEM images and (**e**,**f**) TEM images of MEG-PPV/P3HT blend nanofibers with different PHT contents. Reproduced with permission from Li et al. [21]. Copyright © 2004, WILEY-VCH.

Electrospinning is a versatile method used to produce long, continuous fibers with diameters ranging from tens of nanometers to several micrometers [15]. Figure 2a illustrates the basic setup for the electrospinning process, which consists of key components such as a high-voltage power supply, a syringe pump, a spinneret, and a conductive collector. In the electrospinning process for conjugated polymers, a solution of the polymer dissolved in a suitable solvent is pumped through a spinneret or syringe needle under high voltage, applied between the spinneret and a collector substrate (as depicted in Figure 2a). The electric field exerts a force on the polymer solution, forming a thin, electrified jet that rapidly stretches and thins as it travels toward the collector. During this journey from the spinneret to the collector, the solvent evaporates quickly, leaving behind solid conjugated polymer fibers on the grounded collector. One of the notable advantages of the electrospinning process is its ability to precisely control the fiber diameter, ranging from nanometers to micrometers, by adjusting various parameters such as the applied voltage, liquid flow rate, and spinneret-to-collector distance [15]. For comprehensive reviews of electrospinning methods, please refer to references [15,16].

The electrospinning process finds applications in producing fibers from various materials, including conjugated polymer solutions. Achieving electrospun polymer fibers involves a set of parameters related to the polymer, solvent, polymer solution, processing conditions, and ambient factors [15]. Conducting polymers like polyaniline (PANi) and polypyrrole (PPy) have been electrospun into nanofiber forms. For instance, Chronakis et al. [12] demonstrated the production of polypyrrole nanofibers with diameters ranging from 70 to 300 nm using electrospinning. Bagchi et al. [17] employed an electrospinning coating technique to deposit polypyrrole composites onto optical fibers. MacDiarmid et al. [18] reported PANi nanofibers with an approximate diameter of 139 nm through electrospinning, using a 20 wt% PANi solution in 98% formic acid and a water bath as the collector. In another study, Wu et al. [19] combined the electrospinning method with an in situ solution polymerization process to prepare core-sheath polyaniline (PANi)/polyacrylonitrile (PAN) nanofibers. PEDOT:PSS nanofibers have also been successfully produced via electrospinning, as demonstrated by Huang et al. [20]. They achieved the nanofibers by slightly doping magnesium nitrate as a polymer cross linker to enhance PEDOT:PSS entanglement. The electrospinning process has been modified with a coaxial needle, allowing for the production of nanofibers with a core-sheath structure [21,22]. For instance, Li et al. [21] reported the creation of electrospun nanofibers by blending conjugated polymers such as poly[2-methoxy-5-(2-ethylhexoxy)-1,4-phenylenevinylene] (MEH-PPV) with regioregular poly(3-hexylthiophene) (PHT) using a coaxial two-capillary spinneret, as shown in Figure 2b. Scanning electron microscopy (SEM) and transmission electron microscopy (TEM) images of MEG-PPV/P3HT blend nanofibers are displayed in Figure 2c–f.

#### 2.2.2. Electrohydrodynamic (EHD) Jet Printing

**Figure 3:**
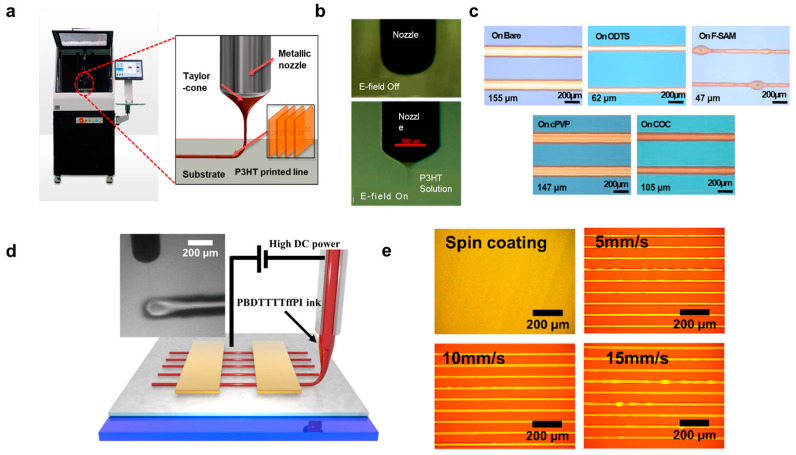
(**a**) A picture of an electrohydrodynamic (EHD) jet printing machine and a printed line of P3HT solution from a metallic nozzle (right). (**b**) Snapshot images of the nozzle during the printing process. (**c**) Optical microscopy images of P3HT lines on different substrates. Reproduced with permission from Jeong et al. [24]. Copyright © 2014, American Chemical Society. (**d**) Schematic illustration of the EHD jet printing setup for PBDTTTTffPT and (**e**) optical microscopy images of the polymer films obtained by spin coating and EHD jet printing at different printing speeds. Reproduced with permission from Li et al. [25]. Copyright © 2022, American Chemical Society.

Electrohydrodynamic (EHD) jet printing stands out as a non-contact printing method, utilizing an electric field between a nozzle and a substrate to pattern high-resolution organic semiconductor fibers. Key characteristics of the printed fibers can be finely tuned by adjusting parameters such as solution concentration, printing speed, nozzle temperature, and substrate surface energy. Furthermore, surface modification of the substrate plays a crucial role in influencing fiber width and P3HT morphology, ultimately impacting charge mobility. For a more in-depth understanding of EHD jet printing, please refer to the comprehensive review paper in [23]. One-directionally oriented fibers using high-molecular-weight semiconductors, like P3HT, have been successfully achieved through the electrohydrodynamic (EHD) jet printing method. For instance, Jeong et al. [24] used EHD jet printing to fabricate microscale P3HT lines for organic field-effect transistors (OFETs), as illustrated in Figure 3a–c. A typical EHD jet printing apparatus is depicted in Figure 3a, where an electric field ranging from 1.8 to 2.0 kV was applied between the metallic nozzle and the substrate. The flow rate of the P3HT solution to the nozzle was maintained at 0.5–0.8 μL/min, resulting in the formation of the characteristic conical shape of the P3HT solution known as a Taylor-cone (Figure 3a). Figure 3b showcases images of the ejection of the P3HT solution before and after the application of the electric field. To ensure a stable printing process and a straight-printed P3HT line, a distance of 1 mm between the nozzle tip and the substrate and a speed of 20–30 mm/min were used. The influence of surface modifications on the resolution of printed lines was also explored, employing F-terminated self-assembled monolayers (F-SAMs) and polymer thin films, as demonstrated in Figure 3c. The bare substrate with the highest surface energy (hydrophilic) resulted in wider lines of the P3HT compared to narrower lines of the P3HT on the F-SAM-modified substrate with the lowest surface energy (hydrophobic). They explained that the width of the P3HT lines increased with increasing surface energy due to the higher surface wettability of P3HT/toluene on the hydrophilic surface. In another study, Li et al. [25] employed EHD jet printing for phthalimide-derived conjugated polymers, specifically PBDTTTffPI, in the fabrication of OFETs and logic gates, as depicted in Figure 3d,e. Furthermore, Chang et al. [26] utilized EHD printing to create microscale PEDOT:PSS-PEO features, expanding the application possibilities of this versatile technique. The smallest width of PEDOT:PSS lines was approximately 27 μm with a nozzle diameter of 100 μm. Finally, Tang et al. [27] harnessed EHD jet printing to produce PEDOT:PSS composite patterns. Using optimized conditions for a stable cone-jet mode, microscale PEDOT:PSS/additive/hardener straight lines for electrodes in OFETs were successfully printed.

#### 2.2.3. Wet Spinning

**Figure 4:**
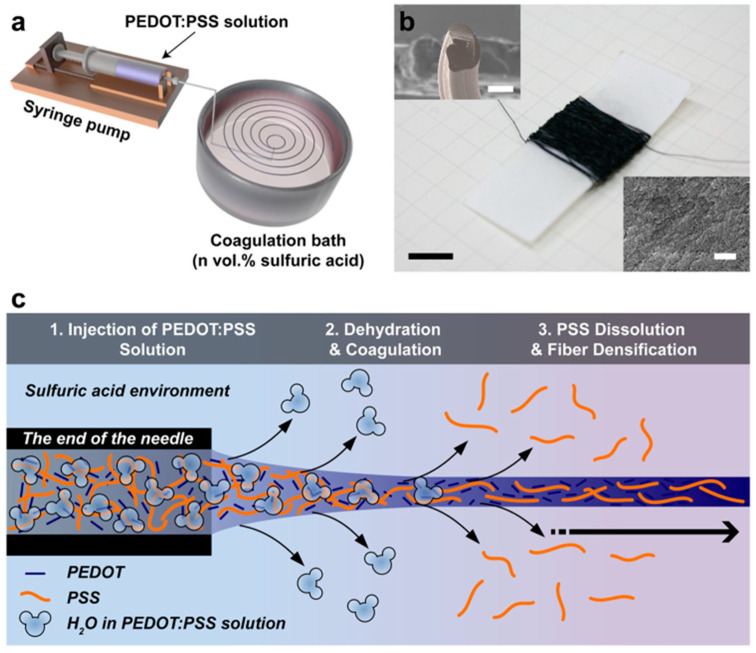
(**a**) Schematic illustration of a wet-spinning process for PEDOT:PSS microfibers. (**b**) A photograph of the spooled microfiber in a paper reel with SEM images of the microfiber cross section (top left) and the microfiber surface (bottom right). (**c**) Mechanism of the formation of PEDOT:PSS microfibers in an acidic coagulation bath. Reproduced with permission from Kim et al. [28]. Copyright © 2018, Springer Nature.

The wet-spinning method is another widely employed technique for producing fibers from conjugated polymers. The process initiates with the dissolution of a conjugated polymer in a suitable solvent, yielding a uniform polymer solution. This solution is subsequently extruded through a spinneret into a chemical bath or coagulation bath containing a non-solvent for the polymer. As the polymer solution enters the coagulation bath, precipitation occurs due to the dilution effect or chemical reactions, leading to the formation of solid fibers. Typically, these fibers are collected using a rotating drum or a similar mechanism. Wet spinning has been successfully employed to produce PEDOT:PSS fibers. For instance, Kim et al. [28] utilized a straightforward wet-spinning process to create conductive PEDOT:PSS microfibers, which were then applied to organic electrochemical transistors (Figure 4). They achieved this by injecting a PEDOT:PSS solution with a solid concentration of 2.0–2.5 wt.% through a syringe needle into an acidic coagulation bath containing varying concentrations (20, 40, 60, 80, or 100 vol.%) of sulfuric acid in water. This resulted in the immediate formation of continuous black microfibers upon injection, as shown in Figure 4a. Figure 4b depicts the continuous PEDOT:PSS fibers characterized by a circular/oval cross section and a rough surface. Notably, the cross-sectional area of the PEDOT:PSS microfibers decreased, while their electrical properties improved with an increasing sulfuric acid concentration in the coagulation bath. The authors proposed the mechanism behind fiber formation in the acidic coagulation bath, as illustrated in Figure 4c. Upon injection of the PEDOT:PSS solution into the coagulation bath, the PEDOT:PSS microfiber coagulates, while any excess PSS chains dissolve in the acid solution, enhancing the crystallinity of PEDOT. In a different study, Feng et al. [29] introduced metal chloride salts into the coagulation bath to expedite the solidification of PEDOT:PSS fibers. Meanwhile, Foroughi et al. [30] employed a reactive wet-spinning approach to produce polypyrrole fibers. In their research, they utilized alginate, a water-soluble polymer derived from marine brown algae, serving as a host fiber containing a pyrrole monomer that was subsequently transformed into the conducting polymer.

#### 2.2.4. Vapor Coating, In Situ Polymerization, and Dip Coating

Here, we describe fibers acting as a template onto which a conjugated polymer is coated. Several methods are used to coat conjugated polymers as follows.

**Figure 5:**
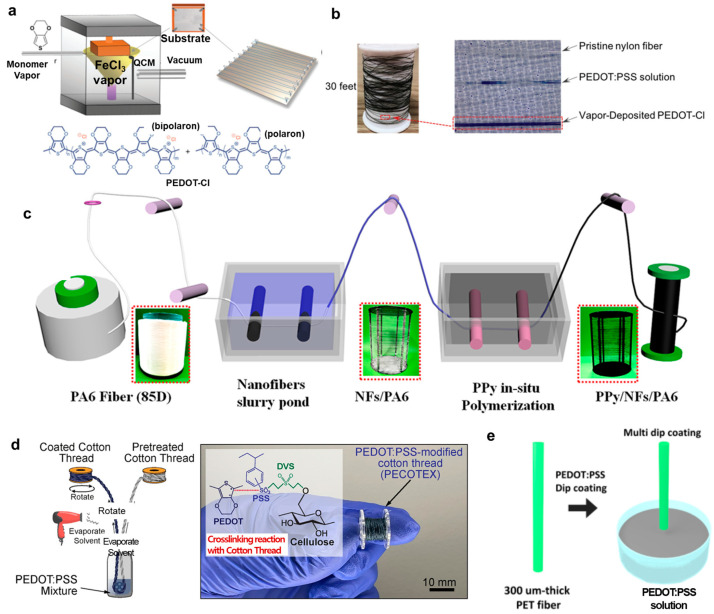
(**a**) Schematic illustration of the vapor coating fiber with the holder and substrate, and the chemical structure of the PEDOT-Cl polymer used for the coating (bottom). (**b**) Optical images of PEDOT-Cl-coated nylon fiber in comparison with dip coating. Reproduced with permission from Zhang et al. [31]. Copyright © 2018, WILEY-VCH. (**c**) The process of making PPy/NFs/PA6 fibers using the in situ polymerization method. Reproduced with permission from Qing et al. [34]. Copyright © 2019, American Chemical Society. (**d**) Schematic drawing of the preparation of PEDOT:PSS-modified cotton thread (PECOTEX) and a photograph of the thread produced on the bobbin (right). Reproduced with permission from Alshabouna et al. [38]. Copyright © 2022, Elsevier. (**e**) An illustration of PET fiber dip coated in PEDOT:PSS solution. Reproduced with permission from Kwon et al. [37]. Copyright © 2018, American Chemical Society.

**Vapor polymerization deposition**: The vapor polymerization deposition can yield a uniform coating of conjugated polymers. Zhang et al. [31] reported a reactive vapor deposition (RVD) method used to conformally coat PEDOT-Cl to nylon monofilament fibers to fabricate OECTs. A schematic illustration of the RVD chamber and substrate holder is shown in Figure 5a. A 1 mm gap between the substrate holder and fibers allowed vaporized reactive species to access all exposed surfaces of the fiber, forming a uniform and continuous surface coating. Thirty feet of fiber with approximately 300 nm thick coating can be coated per deposition cycle. A detailed procedure for RVD can be found in reference [31,32]. Highly doped PEDOT-Cl fibers were obtained through methanol immersion to remove trapped FeCl_3_. A comparison of the uniformity of coating between solution coating and RVD coating is shown in Figure 5b. Hydrophobic nylon monofilament fiber immersed in the PEDOT:PSS aqueous solution can yield a non-uniform coating, while uniform coating can be obtained by RVD. In another study to prepare PEDOT nanofibers, Cetin et al. [33] used a chemical vapor polymerization approach. Chemical vapor polymerization yields a thinner surface coating using small monomer quantities without using solvent [33]. In their work, they first used electrospinning to produce polyacrylonitrile (PAN) nanofibers. The electrospun PAN nanofibers were then dipped into 20 wt% FeCl_3_/ethanol and placed into a glass reactor. Thereafter, they were exposed to EDOT (100 μL) vapor under an active vacuum for 15 s and then under a passive vacuum from 5 min to 24 h. These nanofibers were then washed with methanol and dried under vacuum.

**In situ polymerization**: The in situ polymerization has been used to a great extent for producing polypyrrole fibers. Qing et al. [34] used the in situ polymerization method to prepare PPy/NFs/PA6 filaments. The process of the in situ polymerization is shown in Figure 5c. First, the PA6 filament was immersed in 2 wt% NF suspension. The NFs/PA6 fiber was fully immersed in a mixed pyrrole monomer solution, and the mixed solution with the sample ring was then vibrated gently for 10 min before placing it in the refrigerator for 10–15 min. An oxidizing agent solution consisting of 5-sulfosalicylic acid dihydrate, iron (III) nitrate nonahydrate (the mole ratio was 1:1), and DI water was also placed in the refrigerator. After that, the oxidizing agent solution was added to the mixed solution for about 10–20 min. All the operations were performed in an ice bath for 4 h under continuous stirring. A complete PPy/NFs/PA6 can be obtained after washing and drying in the fume hood. A similar method was used by Wang et al. [35] to prepare PPy/NFs/nylon fibers to fabricate OECTs for lead ion detection and Zhang et al. [36] to coat pyrrole on the surface of MWCNT-modified PA6 fibers to fabricate OCETs. 

**Dip coating method**: The dip coating method has been used to a great extent to prepare conjugated polymers such as PEDOT:PSS and P3HT fibers. This is a cost-effective solution process to coat cylindrical-shaped fibers, which can significantly reduce production costs [37]. In a very recent study, Alshabouna et al. [38] used the dip coating method to prepare PEDOT:PSS-modified cotton thread (PECOTEX) as shown in Figure 5d. The cotton thread was pulled through tubes containing aqueous solutions of PEDOT:PSS using a servo motor before winding on another bobbin after coating. Excess solvent was blow dried before winding on the bobbin to prevent sticking. The optimal speed rotation of the servo motor was controlled to be 3.6 m/min. In another study, Kwon et al. [37] used the dip-coating method to deposit PEDOT:PSS onto PET fibers as shown in Figure 5e. Precleaned fibers were dip coated in a PEDOT:PSS solution at a withdrawal speed of 40 mm/min or 120 mm/min and then annealed for 30 min. The dip-coating method has been also found in other studies, such as PEDOT:PSS on polyamide monofilaments [39], PEDOT:PSS on silk fibers [40], PEDOT:PSS on single cotton fibers [41,42], and PEDOT:PSS-PU composites on PA/lycra knitted fabric [43]. To make P3HT fibers, Kim et al. [44] coated Au microfibers with P3HT and then twisted these fibers to form a field-effect transistor.

## 3. Fiber-Type Conjugated Polymer Transistors

In the fiber-type transistors, there are two main types: fiber field-effect transistors (FETs) and organic electrochemical transistors (OECTs). We will talk about these two transistor types and their integration into textiles below.

### 3.1. Field-Effect Transistors Using Conjugated Polymer Fibers

**Figure 6:**
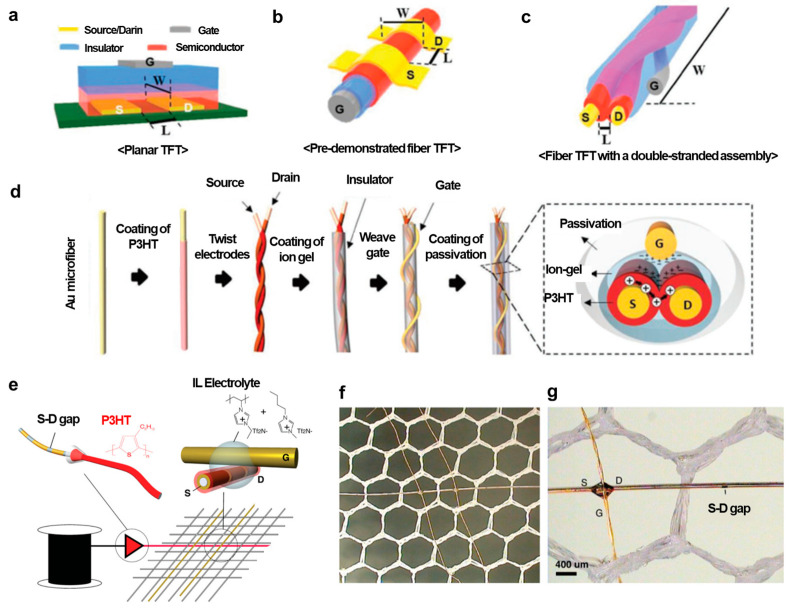
(**a**) Schematic illustration of a planar field-effect transistor on a flat substrate and (**b**) on a conductive fiber. (**c**) Field-effect transistor based on twisted microfibers. (**d**) Schematic illustration of making fiber field-effect transistors using twisted fibers. Reproduced with permission from Kim et al. [44]. Copyright © 2019 WILEY-VCH. (**e**) Schematic diagram of an electrolyte-gated OFET with a prepatterned source−drain on fibers and then coated with PH3T (top left). A junction transistor is formed through an ionic liquid electrolyte (top right) and the weaving of transistors into the fabric using different fibers (bottom). (**f**) Optical microscopy image of two transistors formed along the horizontal fiber. (**g**) A zoom-in optical image of a transistor (left) and a prepatterned source−drain gap (right). Reproduced with permission from Hamedi et al. [46]. Copyright © 2009 WILEY-VCH.

Fiber-type FETs have a similar structure compared to a traditional organic field-effect transistor (OFET), except that the OFETs are usually made on a planar substrate, while fiber-type FETs have a fiber-shaped substrate that is cylindrical. An OFET consists of three major components: three electrodes including a source, drain, and gate; a dielectric layer; an active semiconductor layer. The source and drain electrodes are directly in contact with the semiconductor layer, while the gate electrode is separated from the semiconductor through the insulator layer (Figure 6a). The working principle of this transistor is as follows. A voltage is applied to the gate, controlling the current between the source and drain electrodes. A negative gate voltage applied to an OFET with a p-type semiconductor leads to the formation of an electric field in the dielectric layer. At the side of the dielectric close to the semiconductor, the existence of a negative charge due to the polarization of the dielectric induces the accumulation of positive charge carriers (namely, holes) in the semiconductor close to the semiconductor–dielectric interface, allowing the charge carriers to flow from the source to the drain when placing a voltage on the drain electrode, and the transistor is then on. Fiber-type FETs exhibit similar operating principles to planar OFETs, except that the electric field effect is induced within the fibers. 

There have been some studies demonstrating the use of two or more fibers to fabricate fiber-type transistors together with the use of electrolytes. This creates fiber electrolyte-gated field-effect transistors requiring a low-operating voltage. The working principle of electrolyte-gated field-effect transistors can be explained as follows: When applying a voltage to the gate electrode, the resulting electric field drives cations and anions within the electrolyte to form two electric double layers (EDLs) at the gate electrode–electrolyte and semiconductor–electrolyte interfaces. These EDLs can be considered as extremely thin capacitors with very high capacitance. According to the operating principle of a field-effect transistor, a low-operating voltage can be required. The use of electrolyte gating for transistors also helps to eliminate the problem of an uneven conventional coating of the insulator [45].

An example of a fiber-based electrolyte-gated field-effect transistor using P3HT as the active layer was demonstrated by Hamedi et al. [46], as shown in Figure 6e–g. Two fibers were placed in a cross geometry with an electrolyte bridging the two fibers to create transistors. In one fiber, source−drain Au electrodes were deposited using an evaporator, creating micrometer-scale source−drain gaps. High-purity regioregular P3HT films were dip coated around the gold-coated fibers, which were then woven to create junctions with a second conducting fiber acting as a gate electrode. Finally, a drop of a mixture of solid polymer ionic liquid 1-butyl-3-methyl-imidazolium bis(trifluoromethanesulfonimide) ([bmim] [Tf2N]) and poly(1-vinyl-3-methylimidazolium bis(trifluoromethanesulfonimide) (poly[ViEtIm][Tf2N]) was deposited at the junction to form the electrolyte gated transistor (Figure 6e top right). Multiple transistors could be placed on a single fiber, using traditional fiber production techniques. Figure 6f shows two such transistors created along one single fiber, and Figure 6g shows a magnified image with both the transistor and a visible gap. In another study, Owyeung et al. [45] demonstrated OFET-based P3HT-coated fibers. They reported flexible thread-based transistors using cleanroom-free fabrication processes such as drop casting, drying, and stitching under ambient conditions. No cleanroom process to deposit metal contacts is needed. A linen thread was knotted with source−drain Au wire contacts. Then, the active channel area between the two contacts was coated with P3HT or CNTs. An ion gel was then placed on top of the active channel. Finally, another Au wire used as a gate electrode is in contact with the ion gel. 

For another instance, Kim et al. [44] proposed FETs using P3HT with twisted fibers. Figure 6a illustrates the sophisticated microstructure required to create 1D fiber-type TFTs, where the precise control of microfibers or threads is essential. Channel dimensions, such as channel length (*L*) and width (*W*), in conventional fiber-type TFTs, are primarily limited by the dimensions of microfibers, as shown in Figure 6b. A low drain current can result from the small channel width [44]. Therefore, the researchers designed a semiconductor channel by twisting the source and drain electrode microfibers coated with a polymer semiconductor layer, as depicted in Figure 6c. The overall process for fabricating DSA-fiber TFTs is shown in Figure 6d. The process started with Au microfiber surface modification. Polydopamine (pDA) was used to make the gold surface hydrophilic and improve adhesion with polymer materials. Next, the organic semiconductor P3HT in the range from 60 to 260 nm in film thickness was solution coated using the dye-coating method. The two fibers were then twisted together to form a P3HT channel. Then, an ion gel consisting of poly(vinylidenefluoride-co-hexafluoropropylene) (P(VDF-HFP)) and 1-ethyl-3-methyl imidazolium bis(trifluoromethylsulfonyl) imide ([EMIM] [TFSI]) was drop coated along the twisted microfibers. An outer elastomeric layer was drop coated for passivation, preventing the enclosed microfibers from loosening and protecting them from external substances. In this device configuration, the channel length and width can be easily adjusted by varying the thickness of the semiconductor coating layer and the twist length of the two microfibers. The use of an ion gel as the insulating layer enabled high-performance fiber-type transistors with milliampere-level output currents at low-operating voltages and a good on/off current ratio of 10^5^ with operating gate voltages below −1.3 V. Moreover, the passivation layer on the outermost surface provided durability even during repeated bending and withstood harsh cleaning processes using strong detergent solutions, maintaining nearly constant electrical characteristics.

### 3.2. Fiber-Type Organic Electrochemical Transistors

**Figure 7:**
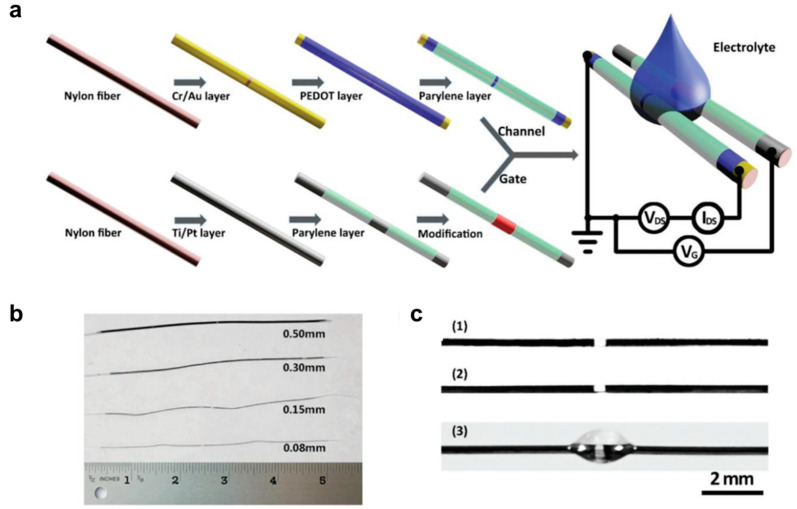
Fiber-type organic electrochemical transistors (OECTs). (**a**) A fibrous OECT integrated into cotton (top) and a schematic diagram of it (bottom). The metal wire on the top image is an Ag gate electrode and the black thread at the bottom is a cotton wire functionalized with PEDOT:PSS. The liquid electrolyte droplet is in contact with both the gate and the cotton wire. (**b**) Photograph of fiber-based devices with different diameters of 0.08, 0.15, 0.3, and 0.5 mm. (**c**) Photograph of a fiber channel before (1) and after (2) coating a PEDOT:PSS layer and (3) with the adhesion of a drop of water to demonstrate the hydrophilic property on its surface. Reproduced with permission from Yang et al. [48]. Copyright © 2019, WILEY-VCH.

Another class of fiber-type transistors is organic electrochemical transistors (OECTs). The structure of these transistors is similar to that of electrolyte-gated field-effect transistors, except for the active channel, which is usually PEDOT:PSS, an ion-permeable polymer. The operating principle of these transistors can be explained as follows: When a voltage is applied to the gate, charge induction occurs at the junction, and the induced charge flows between the source and drain fiber ends, generating the electrical response. The gate voltage allows ions from the electrolyte to penetrate the semiconductor layer, generating charge carriers through electrochemical reactions (oxidation and reduction reactions). One of the biggest advantages of fiber-type OECTs is that their operation voltage does not rely on the thickness of the dielectric, but rather on the electrochemical potential of the system, which is approximately one volt [47]. Additionally, the simple fabrication process and simple structure make fiber-type OECTs a potential candidate for textile applications. Hamedi et al. [39] (2007) used PEDOT:PSS-coated fibers to make the OECTs junction type. Two polyamide monofilaments were coated with PEDOT:PSS solution. The two coated filaments were manually placed into a cross geometry. At the cross junction, a drop of solid polymer was placed to create a complete OECT. The same group also demonstrated the PEDOT:PSS fiber OECTs junction type [40]. PEDOT:PSS-stained silk fibers were placed cross to each other, and at the cross junction a drop of ionic liquid (IL) 1-butyl-3-methylimidazolium bis(trifluoromethanesulfonim ide) ([bmim][Tf2N]) and the corresponding polymer ionic liquid (PIL) poly(1-vinyl-3-methylimidazolium) bis(trifluoromethane sulfonimide) (poly[ViEtIm][Tf2N]) was added. Yang et al. [48] used metal/conductive polymer multilayer electrodes on nylon fiber to make OECTs, as shown in Figure 7a. Nylon fiber was coated with a Cr/Au layer by magnetron sputtering and then a PEDOT:PSS layer. A Parylene layer was used to passivate the conductive layer. In another nylon fiber, a Ti/Pt layer was first coated, and then a Parylene layer was coated except for the area of the gate electrode. These two nylon fibers were in contact with an electrolyte, phosphate-buffered saline (PBS) solution, to form a complete OECT. Figure 7b,c shows the nylon fibers with different diameters and hydrophilic levels on their surfaces.

### 3.3. Integration of Fiber-Type Transistors into Textiles

**Figure 8:**
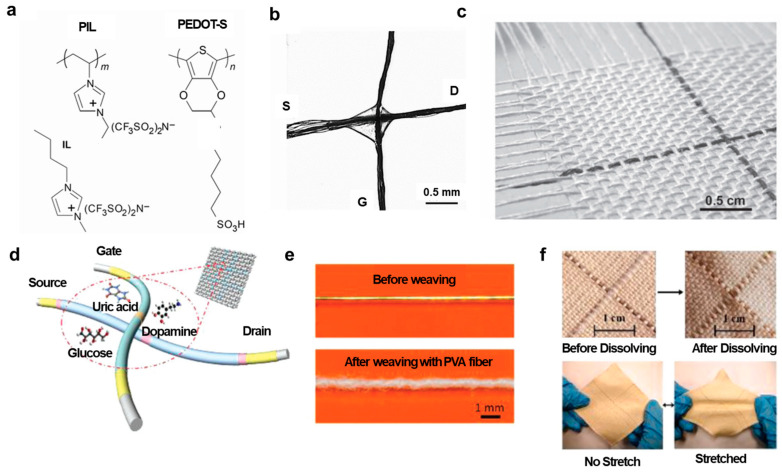
(**a**) The chemical structure of the imidazolium-based ionic liquid:polymer ionic liquid (IL:PIL) electrolyte mixture [bmim][Tf2N]:poly[ViEtIm][Tf2N] and the conjugated polyelectrolyte dye PEDOT:PSS. (**b**) Microscopy image of a silk fiber-based ECT device with a source (S), drain (D), and gate (G). (**c**) A photograph of a basket weaving fabric manually woven with pristine as well as PEDOT:PSS-stained silk threads. Reproduced with permission from Muller et al. [40]. Copyright © 2011, WILEY-VCH. (**d**) Schematic diagram of an OECT sensor obtained by weaving fiber-based devices with cotton yarn. (**e**) Photographs of fibers before and after weaving with PVA-protecting yarns. (**f**) Fabric devices before and after the removal of PVA on fibers. Reproduced with permission from Yang et al. [48]. Copyright © 2019, WILEY-VCH.

Fiber-type transistors have distinguishing features of their fiber-type shape when compared to conventional transistors. They can be woven into fabrics or textiles, enabling the development of smart textiles and wearable electronics. There are two common ways to integrate fiber-type transistors into electronic textiles [8]. The first is to embed electronic devices such as transistors directly in textiles or fabrics. The second is to weave the electronic fibers into textiles. Hamedi et al. [46] used different functional fibers on reels and weaved these fibers into fabrics to form transistors (Figure 6e–g). Muller et al. [40] demonstrated PEDOT:PSS OECTs woven into the fabric (Figure 8a–c). Figure 8a shows materials including PEDOT:PSS-stained silk fibers, ionic liquid (IL) 1-butyl-3-methylimidazolium bis(trifluoromethanesulfonim ide) ([bmim][Tf2N]), and polymer ionic liquid (PIL) poly(1-vinyl-3-methylimidazolium) bis(trifluoromethane sulfonimide) (poly[ViEtIm][Tf2N]), which are used to fabricate an OECT. Figure 8b shows an OECT with two PEDOT:PSS fibers in a simple cross-junction geometry that is bridged by a 1:1 mixture of the ionic liquid and the polymer ionic liquid. Figure 8c shows PEDOT:PSS-stained silk fibers manually woven into fabrics. These fibers remain unaffected by rough handling, such as threading through a sewing needle and stitching. Additionally, Yang et al. [48] weaved fiber-type PEDOT:PSS OECTs with cotton yarns using a conventional weaving machine (Figure 8d–f). Figure 8d shows PEDOT:PSS OECTs as both warp and weft in the weaving process, allowing for precise control of the distance between the channel and the gate. However, the weaving process poses a challenge as the devices are frequently rubbed by yarns, degrading their performance. Therefore, a protective layer, polyvinyl alcohol (PVA) yarn, was used to apply to the fibers using a braiding machine. Figure 8e shows the fibers before and after covering the PVA layer. The PVA layer can be easily dissolved in water after weaving. Figure 8f shows a fabric woven with two PEDOT:PSS transistors. This fabric was described as highly flexible, stretchable, and capable of fully recovering its shape even after extensive stretching. 

## 4. Application of Fiber-Type Transistors in Sensors

Fiber-type transistors have been applied in various fields, such as memory, energy storage, chemical and biosensors, light-emitting devices, and solar cells. In this review, we mainly focus on chemical sensors to detect glucose and other chemical molecules, and physical sensors including tactile sensors. All the sensors described in this review are summarized in Table 1.

### 4.1. Chemical Sensors

#### 4.1.1. Glucose Sensors

**Figure 9:**
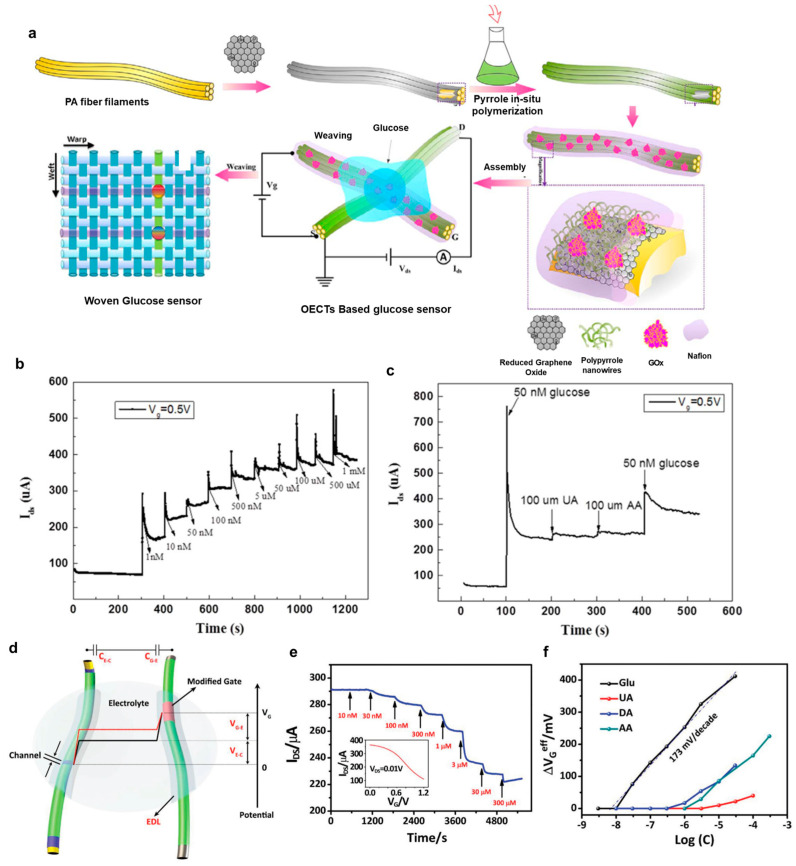
(**a**) Schematic diagram of a glucose-sensing system utilizing fiber-based organic electrochemical transistors (OECTs) with an active layer composed of PPy nanowires and rGO. (**b**) Amperometric responses of the source−drain current response observed in the FECT sensor upon the addition of varying glucose concentrations. The applied gate voltage (V_g_) is 0.5 V, and the drain−source voltage (V_ds_) is set to −2 V. (**c**) Amperometric responses of the biosensor when exposed to glucose (50 nM), UA (uric acid, 100 µM), and AA (ascorbic acid, 100 µM) in a phosphate-buffered saline (PBS) solution. The gate voltage (V_g_) is set to 0.5 V. Reproduced with permission from Wang et al. [52]. Copyright © 2017, Elsevier. (**d**) Schematic diagram of a fiber-based organic electrochemical transistor (OECT) operating within an electrolyte, with potential variations occurring across two electric double layers (EDLs). (**e**) Current responses of the device and (**f**) the corresponding changes in effective gate voltage as a result of introducing glucose (Glu) at various concentrations, including UA, DA (dopamine), and AA. The drain−source voltage (V_ds_) is set to 0.01 V, and the gate voltage (V_g_) is set to 0.6 V. Reproduced with permission from Yang et al. [48]. Copyright © 2018, WILEY-VCH.

Glucose sensors are vital medical devices that provide their users with important information about sugar levels in the blood, and are particularly significant for managing diseases such as diabetes mellitus [49]. Notably, glucose levels can be non-invasively monitored in bodily fluids like saliva, tears, and sweat [50]. Among these fluids, sweat emerges as a particularly accessible and promising medium for glucose sensing [51]. Therefore, there is substantial research interest in developing glucose sensors using fiber-type transistors with a focus on their applications in non-invasive and continuous glucose monitoring. These sensors can be seamlessly integrated into fabrics and clothing, efficiently collecting sweat from the skin for analysis. A research team from Wuhan University has successfully developed glucose sensors-based organic electrochemical transistors (OECTs) using a combination of polypyrrole nanowires and reduced graphene oxide (rGO) [52]. They achieved this by integrating polypyrrole into rGO sheets through a straightforward in situ polymerization process, resulting in the creation of a hybrid active layer for fiber-based organic thin-film transistors. This PPy/rGO composite was applied to a polyamide (PA) filament, and a glucose sensor was developed by immobilizing glucose oxidase on the filament with Nafion (Figure 9a–c). Figure 9a illustrates the schematic representation of a glucose sensor based on OECTs, featuring the PPy/rGO composite as the active layer. The source and drain electrodes and the gate electrode filament were fabricated using PPy/rGO/PA6 and PPy/rGO/PA6/Gox/Nafion, respectively. These electrodes were configured in a simple cross-junction arrangement, with an electrolyte gel interface at the junction point of the filaments. Finally, large-area textile-based sensors were woven into fabrics using functionalized coated filaments (Figure 9a bottom left). The chemical reactions occurring near the gate electrode are outlined below:

$$\begin{array}{l} Glucose+GOx\left(ox\right)\to D-glucono-1,5-lactone+GOx\left(red\right)\\ GOx\left(red\right)+O_{2}\to GOx\left(ox\right)+H_{2}O_{2}\\ {LiClO}_{4}+4H_{2}O_{2}\to 4H_{2}O+4O_{2}+LiCl(8e^{-}) \end{array}$$

Glucose undergoes catalysis by glucose oxidase (GOx), which is attached to the surface of the gate electrode. This catalytic process generates hydrogen peroxide (H_2_O_2_) and enables electron transfer near the gate electrode’s surface [53]. Consequently, this alteration in the effective gate voltage applied to the transistor affects the channel current. Since the concentration of H_2_O_2_ is directly correlated with the concentration of glucose, H_2_O_2_ is commonly utilized for detecting and quantifying glucose levels [54,55,56].

Figure 9b depicts the amperometric response observed over time when glucose was introduced into the organic electrochemical transistors (OECTs). During these experiments, the gate and drain−source electrode potentials were maintained at 0.5 V and −2 V, respectively. Notably, when the glucose concentration increased from 1 nM to 1 μM, a substantial and rapid rise in current occurred within just 0.5 s. The current exhibited stepwise increments and took approximately 5 s to stabilize when different glucose concentrations were introduced. At lower concentrations, the current increased swiftly, but beyond 1 mM, the response curve tended to plateau. Their devices exhibited a linear current response with a 0.9792 correlation coefficient (R) and a sensitivity of 0.773 normalized current response (NCR) per decade. The low-glucose concentration detection can be attributed to the strong electron-accepting properties of perchlorate ions and the unique structure of reduced graphene oxide (rGO), which offers a high surface area-to-volume ratio, enabling greater enzyme loading and facilitating efficient charge transfer [52]. The selectivity property of their devices was also investigated using 100 μM ascorbic acid, 100 μM uric acid, and 50 μM glucose (Figure 9c). When 50 nM glucose was added, a clear current response was observed, whereas there was no noticeable current response when 100 mM concentrations of ascorbic acid or uric acid were introduced. This distinct difference in responses between glucose and the interfering substances demonstrates the high specificity of the PPy/rGO-based OECTs for glucose, even in the presence of potential interferences. For reproducibility, they prepared five different transistors to test the same glucose concentration. They achieved a uniform response among all devices, with a mere relative standard deviation of 7.1%, suggesting the impressive reproducibility of their glucose sensors. Furthermore, the practicality of these sensors was tested using real rabbit blood samples. Notably, the glucose concentration readings from their sensors closely aligned with those obtained through spectrophotometry, exhibiting only a 10% difference in relative standard deviation (RSD), even at very low-glucose concentrations. This suggests the suitability of their OECT glucose sensors for real-world applications.

Another example of glucose sensors based on conductive polymer PEDOT:PSS organic electrochemical transistors (OECTs) has been demonstrated by Yan’s group at The Hong Kong Polytechnic University [48] (Figure 9d–f). These sensors were fabricated by coating nylon fibers with PEDOT:PSS channels and incorporating them into fabric materials as shown in a previous figure, Figure 8d–f. For the source and drain electrodes, highly conductive multilayer films comprising Cr/Au/PEDOT:PSS were applied to the fibers. These devices exhibited remarkable flexibility and durability, making them suitable for various chemical and biological sensing applications. A schematic of their fiber-based OECT sensor is shown in Figure 9d with two fibers simply connected by an electrolyte. In Figure 9d, the sensing mechanism of the fiber-based OECT sensor is also depicted. A gate voltage is applied to two electric double layers (EDLs) on the surfaces of the channel and gate, respectively. Interaction between the analyte and the gate or channel alters the potential drop at an EDL, resulting in a change in the effective gate voltage [55,57]. The fiber-based OECTs were employed as glucose sensors by modifying the Pt gate electrodes with a composite consisting of the enzyme glucose oxidase (GOx), chitosan (CHIT), and graphene flakes on their surfaces [54]. Figure 9e illustrates the responses of a fiber-based glucose sensor to different glucose concentrations in a phosphate-buffered saline (PBS) solution. The device’s channel current decreases as the glucose concentration increases, achieving a remarkably low detection limit (LOD) of 30 × 10^−9^ M. This glucose concentration range is the physiological glucose level in human body fluids such as saliva, suggesting great potential for non-invasive glucose sensing applications. In terms of selectivity, Figure 9f shows that the sensor has a detection limit for glucose nearly two orders of magnitude lower than that for three common interferences, namely, uric acid (UA), dopamine (DA), and ascorbic acid (AA). The selectivity can be explained due to the presence of GOx and the modified polymer CHIT on the fiber gate electrode, acting as an effective barrier against interferences [58]. In a PBS environment, the negatively charged CHIT layer can repel anionic interferences like UA and AA due to electrostatic forces. The authors then weaved their devices using cotton yarns on a conventional weaving machine (see Figure 8f) and subsequently tested them with various glucose concentrations in a PBS solution. Notably, the devices exhibited a detectable response at a concentration as low as 30 × 10^−9^ M, similar to the detection limit of the devices before the weaving process. To illustrate the device’s practical application, they incorporated a fabric-based OECT into a diaper and used it to monitor glucose levels in artificial urine absorbed by the diaper. The device detected the glucose concentration as low as 3 × 10^−3^ M and showed good selectivity when 1 × 10^−3^ M uric acid was added. Also, note that the diaper underwent repeated bending and stretching during measurements. The device, however, exhibited minimal impact on its performance, suggesting the excellent stability of the device’s performance. 

#### 4.1.2. Ionic Concentration Sensors in Human Sweat

**Figure 10:**
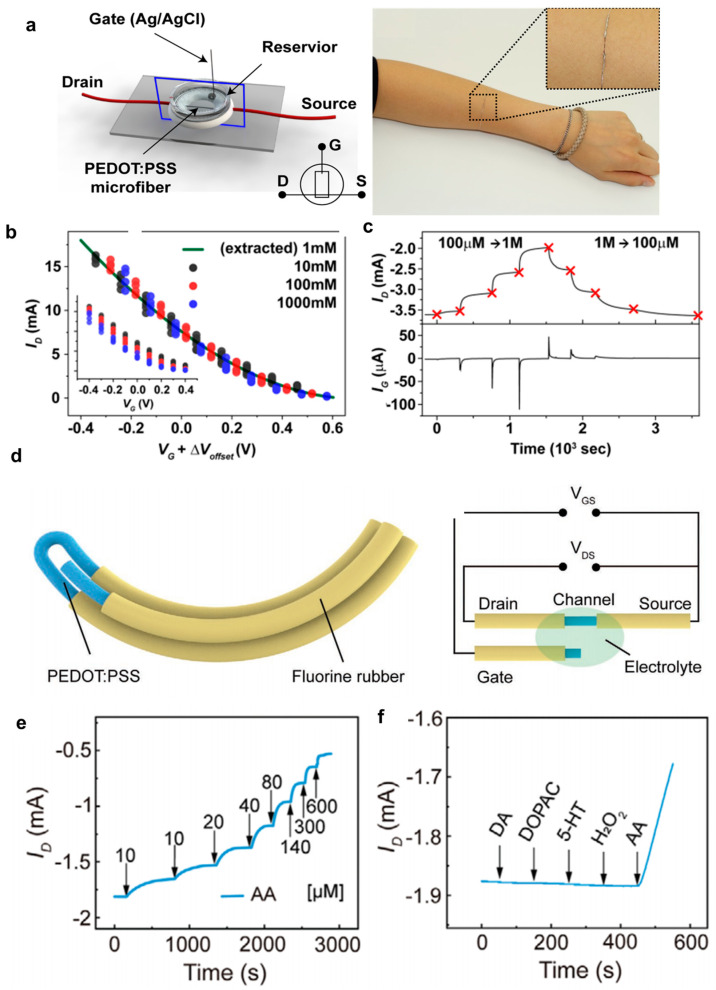
(**a**) Illustration of a PEDOT:PSS microfiber organic electrochemical transistor (OECT) equipped with three terminals designed for the detection of cation concentrations (left) and a photograph of a single-strand channel PEDOT:PSS microfiber OECT directly attached to human skin (right). (**b**) Offset voltage-compensated (V_g_ + ΔV_offset_) transfer curves recorded for a PEDOT:PSS microfiber OECT. This specific OECT features characteristics including 20% H_2_SO_4_ coagulation, a cross-sectional area of 12.3 × 10^–5^ cm^2^, and a conductivity of 318 S/cm. These measurements were taken while the OECT was immersed in NaCl solutions with concentrations of 10, 10^2^, and 10^3^ mM. To calibrate the gate voltage relative to the 1 mM condition (indicated by the green line), a theoretical ΔV_offset_ (0.059 V/dec; 59, 118, and 177 mV) was added. The inset displays the uncompensated transfer curves. (**c**) Tracking the temporal changes in both drain (upper) and gate currents (lower) for the same PEDOT:PSS microfiber OECT when exposed to NaCl solutions of varying concentrations, spanning from 10^–1^ to 10^3^ mM and from 10^3^ to 10^–1^ mM. Reproduced with permission from Kim et al. [28]. Copyright © 2018, Elsevier. (**d**) Schematic of the circuit diagram outlining the PF-OECT (Polymer Fiber Organic Electrochemical Transistor). (**e**) Drain current response was observed during the sequential addition of ascorbic acid (AA) into the electrolyte solution. (**f**) Amperometric response in drain current upon introducing substances including 10 nm dopamine (DA), 10 nm 3,4-dihydroxyphenylacetic acid (DOPAC), 10 nm serotonin (5-HT), 10 μm hydrogen peroxide (H_2_O_2_), and 100 μm ascorbic acid (AA). Reproduced with permission from Feng et al. [64]. Copyright © 2023, Wiley-VCH.

A research team at the Gwangju Institute of Science and Technology has developed a wearable sweat sensor based on the conductive polymer PEDOT:PSS [28]. They have designed microfiber-based organic electrochemical transistors (OECTs) for use as channel dimension-independent wearable sensors to measure ion concentrations in human sweat (Figure 10a–c). These OECT devices without a substrate were created by carefully considering the acidity of the coagulation medium’s impact on OECT characteristics. This impact was examined in terms of the device’s response to various concentrations of aqueous ionic solutions in contact with the channel layer and the Ag/AgCl gate electrode. A schematic diagram of a microfiber-based OECT with three terminals can be seen in Figure 10a (left). A PEDOT:PSS microfiber was connected with copper wires using Ag epoxy for source and drain connections. In the electrical measurement, the source and drain electrodes of the microfiber OECT were connected to a source meter, and the current variation was recorded at V_D_ = −0.1 V. Meanwhile, the gate bias was applied to the Ag/AgCl gate electrode immersed in the aqueous electrolyte reservoir (containing NaCl). In Figure 10b, the hydrophobic PEDOT:PSS chains were effectively doped by the hydrophilic PSS chains, allowing small ions to move into or out of the PEDOT:PSS network in the presence of a gate bias and surrounding aqueous ions [59,60]. Consequently, the PEDOT:PSS chains could be de-doped (or doped) by positive (or negative) gate bias and/or high (or low) ion concentration [61]. This modulation of the source-to-drain current through the PEDOT:PSS microfiber channel led to a reduction in drain current and an on/off current ratio of 10^2^ when the gate bias was swept from −0.4 to 0.4 V at a fixed drain voltage of −0.6 V, particularly in a 100 mM NaCl solution. The transfer curve shifted more negatively with higher salt concentrations, indicating that higher small ion concentrations could more effectively interrupt the doped state. According to the authors, they achieved a sensitivity of 01.6/dec for their PEDOT:PSS OECT devices. The time-dependent drain and gate currents were monitored when the NaCl concentration in the reservoir was altered, as shown in Figure 10c. Despite keeping the gate and drain voltages fixed at 0 V and −0.1 V, respectively, to maintain the OECT device in the linear region, the reservoir solution was replaced with fresh solutions of designated NaCl concentrations (ranging from 10^−1^ to 10^3^ mM). As a result, the negative drain current immediately increased upon adding a more concentrated NaCl solution (from 10^−1^ to 10^3^ mM) and decreased when a less concentrated NaCl solution was introduced (from 10^3^ to 10^−1^ mM). Additionally, the migration of ions between the gate electrode and the PEDOT:PSS layer could be monitored by measuring I_G_ when the reservoir solution was replaced. For practical applications, the authors attached their microfiber-based OECT device to an arm (Figure 10a right), suggesting that this fiber-type ion sensor can be embedded into clothes due to its small size and flexibility even with its small diameter of 10–100 µm. They also demonstrated the practicality of employing substrate-free OECTs made from single strands in wearable electronics for human use. These devices were employed to sense the total small ion concentration in PBS, artificial sweat, and human sweat samples. The cation concentrations measured with their OECT sensor for the PBS and artificial sweat samples matched well those obtained from ion chromatography inductively coupled plasma (IC-ICP) mass spectroscopy with only a 3% error. However, the human sweat showed a relatively large error of 10%. The reason is that their OECT-based sensors can only detect monovalent small cations (Na^+^, K^+^), while the IC-ICP can detect all cations, not only monovalent cations but also divalent cations (Ca^2+^, Zn^2+^, Cu^2+^, Fe^2+^, Ni^2+^). The authors additionally highlighted the exceptional stability of the device following repeated measurements of cation concentrations after washing it out with a 1 mM NaCl solution.

Another interesting example of using fiber-based OECT for the detection of the saline concentration in human sweat was demonstrated by Tarabella et al. [41]. The fiber-based OECT consists of a cotton-soaked PEDOT:PSS thread channel, an Ag wire gate parallel to the channel, and a drop of liquid electrolyte to connect the thread channel and the wire gate. They investigated the suitability of fiber-based OECT for the physiological range of human sweat (2 × 10^−2^ to 8 × 10^−2^ M). They detected the physiological range of chloride in the sweat (30–60 mM), suggesting that their fiber-based OECT sensors are suitable for healthcare and fitness applications. The same group also developed cotton-based OECT sensors to detect adrenaline concentrations in human sweat [62]. A cotton yarn was functionalized with PEDOT:PSS for the channel and a Pt wire was used as the gate electrode. The researchers found that by using different gate electrodes such as Ag wires they could independently detect saline concentration and adrenaline concentration in real human sweat. In a recent study, the researchers demonstrated a new textile-based PEDOT:PSS OECT with ion-selective membranes added to detect selectively electrolytes in human sweat [63]. Their devices showed excellent selectivity to sodium, potassium, and calcium ions. The electrolyte concentration of the electrolytes that the sensors can detect was 10^−5^ to 1 M, which is the concentration range found in human sweat. This confirms the ability of their OECT to be integrated into clothes for real-time monitoring of electrolytes in sweat.

#### 4.1.3. Ascorbic Acid Sensors

Ascorbic acid (AA) is widely known as vitamin C with primary functions as an antioxidant, protecting cells from the harmful effects of reactive oxygen species [65]. AA is also a neuromodulator in the central nervous system that is linked to neurological diseases such as epilepsy [64,66]. A research team from Fudan University developed ascorbic acid sensors based on all polymer fiber organic electrochemical transistors (PF-OECT) (Figure 10d) [64]. To address the sensitivity reduction caused by rigid metal and carbon fiber-based electrodes and protein adhesion to the electrode surface in conventional electrochemical transistors, they used PEDOT:PSS fibers to create PF-OECT. These PF-OECTs had a soft, brain-tissue-like Young modulus and minimized protein adhesion due to the high hydrophilicity of PEDOT:PSS. Figure 10d shows the structure of a PF-OECT. A PF-OECT was manufactured with PEDOT:PSS surrounded by a fluorine rubber insulator and channel formation involved bending the fiber with insulating fluorine rubber at both ends. Another fiber was introduced to serve as the gate electrode. Typically, when a positive gate voltage was applied, ascorbic acid underwent oxidation at the gate surface, resulting in a significant reduction in channel resistance and reduced drain current [48,55,57]. Figure 10e demonstrates the sensor’s sensitivity through the continuous addition of ascorbic acid. The result reveals a linear relationship between the change in drain current and ascorbic acid concentration within the range from 10 to 1200 µM with excellent reproducibility. The relationship yielded a high sensitivity of 0.587 ± 0.017 mA (lg([AA] M^−1^))^−1^. Additionally, since the gate voltage required for ascorbic acid oxidation was very low compared to other electroactive substances, a gate voltage of around 0.2 V exhibited excellent selectivity (Figure 10f). There is a significant current change in the OECT sensors when exposed to ascorbic acid, even in the presence of interferences at similar physiological concentrations. The research team also evaluated PF-OECT, focusing on its flexibility, stability, and resistance to biofouling. When the polymer fiber was subjected to simulated in vivo conditions, it showed an exceptional level of flexibility. Remarkably, the PF-OECT maintained its performance consistently under dynamic deformations in a physiological environment, even after being immersed in artificial cerebrospinal fluid at 37 °C for 14 days. Regarding its anti-biofouling characteristics, the PF-OECT demonstrated stability, with only minor fluctuations observed before and after exposure to BSA solutions for 14 days. Finally, the researchers implanted PF-OECTs into the brains of mice for in vivo testing. These devices effectively detected the presence of ascorbic acid, displaying high selectivity over other chemicals. Furthermore, they remained stable throughout the 14-day testing period, suggesting their potential as durable implantable sensors.

Another example of fiber-based OECTs for ascorbic acid sensors has been reported by Fang et al. [67]. Nylon fiber was covered with PEDOT:PSS to serve as a channel, and a CNT fiber gate was placed next to the channel to form a transistor structure. The CNT fiber without modification has catalytic activity for ascorbic acid. The coaxial fiber OECT showed a linear response with the concentration of ascorbic acid in the range from 0.1 to 1 mM, which is close to the concentration of ascorbic acid in human tissue. The sensitivity was 12.78 mA/decade, and the limit of detection was 1 µM. For real applications, the researchers implanted the coaxial fiber OECT with an unmodified CNT fiber gate into the deep brain of mice. The device impressively exhibited strong selectivity, accurately detecting ascorbic acid in the presence of interfering substances like dopamine and glucose. For the biocompatibility of the coaxial fiber OECT, they found minimal inflammation and excellent compatibility of their devices with the biological environment. An interesting point here is that they can use a modified CNT gate with an electron transfer agent, tetrathiafulvalene (TTF), and glucose oxidase (GOx) to easily detect glucose.

#### 4.1.4. Dopamine Sensors

**Figure 11:**
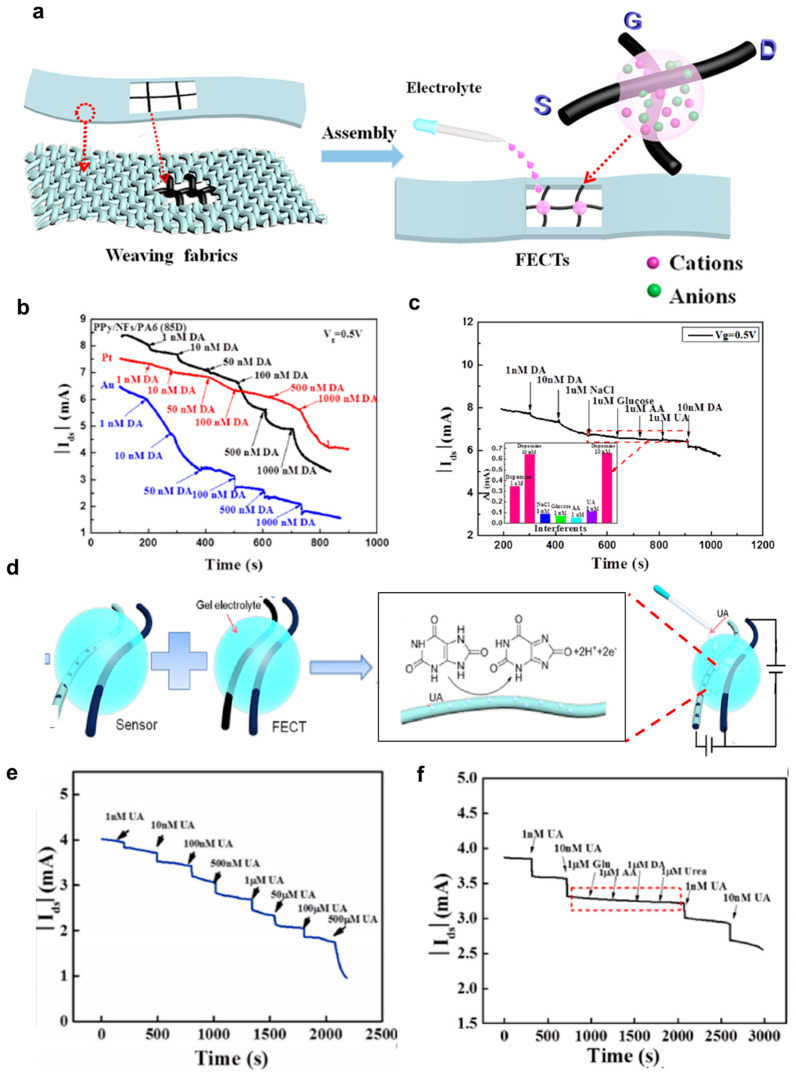
(**a**) Integration process of woven fiber organic electrochemical transistors (FECTs) and wearable dopamine (DA) sensors. (**b**) Amperometric response of FECTs featuring gate electrodes made of PPy/NFs/PA6, Pt, and Au to dopamine concentrations ranging from 1 to 1000 nM in a PBS solution. (**c**) The amperometric response observed in the FECT sensor upon introducing substances, including 1 nM dopamine, 10 nM dopamine, 1 μM NaCl, 1 μM glucose, 1 μM ascorbic acid (AA), 1 μM uric acid (UA), and 10 nM dopamine in PBS at a gate voltage (V_g_) of 0.5 V. Inset: bar chart displaying normalized amperometric current in relation to interfering substances. Reproduced with permission from Qing et al. [34]. Copyright © 2019, American Chemical Society. (**d**) Schematic depiction detailing the fabrication process of FECTs and biosensors. (**e**) Amperometric responses of sensors integrated into the fabric upon the introduction of uric acid (UA). (**f**) Relative current responses of MIPA/PEDOT/carbon fiber gate electrodes to 1 nM UA and 10 nM UA in the presence of various interfering substances (glucose, dopamine, urea, and uric acid) at 1 μM. Reproduced with permission from Tao et al. [76]. Copyright © 2022, American Chemical Society.

Dopamine (DA) is a vital neurotransmitter that holds a central role in various functions within the central nervous system [34,68]. Dysregulation of dopamine is notably associated with two prominent diseases: Parkinson’s syndrome, which is caused by dopamine deficiency, and Alzheimer’s disease, which is linked to dementia [34,69]. Organic thin-film transistors (OTFTs) have gained significant attention for their utility in dopamine detection, primarily owing to their advantages of being cost-effective, easily manufacturable, and highly portable [70,71,72,73]. Organic electrochemical transistors (OECTs), especially fiber-based OECTs have recently emerged as promising dopamine sensors due to their exceptional attributes, including high sensitivity, rapid response times, low detection limits, and remarkable flexibility [34,48,52,56,74,75]. These properties are largely attributed to their larger surface-to-volume ratio. Qing et al. [34] developed dopamine sensors based on fiber-based wearable organic transistors (Figure 11a). They developed fiber-based organic transistors with superior performance compared to conventional transistors. The developed fiber transistors were used as dopamine sensors, utilizing the twisted structure of PVA-co-PE nanofibers (NFs), which provided fast response times, long-term stability, and high productivity. Additionally, the 3D polypyrrole structure induced by NFs enabled the production of high-performance dopamine sensors. Compared to metal gate electrode-based organic fiber transistors, fiber gate electrodes exhibited high initial channel current values, enabling the sensing of dopamine at low concentrations (1 nM) and providing high selectivity. PA6 filaments were adjusted in size in NFs slurry, followed by surface conductivity treatment with PPy to produce PPy/NFs/PA6 fiber transistors. Since these components were manufactured using a commercial weaving machine, they can be designed with various textile textures for diverse circuit configurations and efficient mass production. These two conductive fibers were configured in a cross-linked form, with source−drain fibers separated from gate fibers and a transparent gel electrolyte at the center (Figure 11a). The transfer curve of the fabricated component showed that the device turned off at a gate voltage of 3.2 V, and its performance was tested at a gate voltage of 0.5 V and a drain voltage of −3 V, where the device functioned sufficiently. Initially, the performance of Pt-based and Au-based sensors was compared (Figure 11b). PPy/NFs/PA6 fiber-based devices exhibited the highest initial current value among the three components, as well as the fastest response time and high sensitivity of 47.28 NCR per decade and a correlation coefficient (R^2^) of 0.98085. Furthermore, PPy/NFs/PA6 OECT demonstrated high selectivity performance with the presence of interfering substances including NaCl, uric acid, ascorbic acid, and glucose (Figure 11c). Their devices also exhibited excellent reproducibility, especially when the dopamine concentration is as low as 1 nM.

#### 4.1.5. Uric Acid Sensors

Uric acid is the primary antioxidant and the ultimate byproduct of purine nucleosides, adenosine, and guanosine catabolism [76,77]. The high level of uric acid in body fluids can lead to diseases such as gout, cardiovascular disease, kidney stones, and some metabolic syndromes [65]. Hence, the measurement of uric acid in human urine is crucial for early detection and monitoring of potential disease symptoms. Organic electrochemical transistors (OECTs), especially fiber-based OECTs have gained attention for sensing applications due to their adaptable design, low-operational voltage, and inherent signal amplification capabilities [48,76,78,79]. They offer a relatively large specific surface area, easy integration into fabrics, and seamless incorporation into textiles, providing exceptional sensitivity for various sensing applications. Recently, Tao et al. [76] from Wuhan Textile University conducted a study on synthesizing fiber-based transistors with poly(3,4-ethylenedioxythiophene) (PEDOT) nano-cluster structures via a reverse microfluidic method and utilized them as uric acid sensors (Figure 11d) [76]. First, cotton fiber was reduced with hydrazine hydrate in a gaseous environment to create graphene oxide-coated cotton fiber. Subsequently, rGO/cotton fiber was synthesized through a forward microfluidic method and used as the channel and gate electrode. To create the gate electrode for uric acid sensing, PEDOT-doped carbon fiber was prepared using a similar process. Figure 11d shows the formation of the OECT sensors with two conductive fibers placed in parallel with a distance of 0.5 mm and drops of gel electrolyte connecting the two fibers. The PEDOT/carbon fiber was subjected to electrochemical polymerization with dopamine monomer and uric acid template in pH 7 PBS buffer electrolyte under the voltage range from −0.6 V to 0.6 V and a scan rate of 30 mV/s for 20 cycles. The resulting sample was prepared by removing the uric acid template with a solution of acetic acid/methanol (1:9 ratio). PEDOT/rGO/cotton fiber was sewn onto fabric as source and drain electrodes, while MIP-doped electrodes were used as the gate electrode. The fabricated component was tested with uric acid concentrations ranging from 1 nM to 500 μM, demonstrating changes in current with varying concentrations (Figure 11e). The devices exhibited a sensitivity of 100 μA per decade with a 0.97143 correlation coefficient (R). There was negligible current change after adding successively 1 μM of ascorbic acid, 1 μM of urea, and 1 μM of glucose to the sensor (Figure 11f), confirming the high selectivity of the OECT sensors toward uric acid detection. The high selectivity of the MIP layer can be explained due to the formation of covalent or non-covalent bonds between uric acid molecules and the MIP polymer.

Hao et al. [80] also reported molecularly imprinted polymer-based OECT sensors for uric acid and adrenaline. Here, they used a viscose yarn modified with a multi-walled carbon nanotube (MWCNT) and then coated with PEDOT:PSS for the channel. A carbon fiber yarn modified by a molecularly imprinted polymer (MIP) was used as a gate. These two yarns were placed in parallel and were connected by a gel electrolyte to complete a viscose yarn-based OECT. Their devices exhibited a high transconductance of 6.7 mS, a fast response time of 2 s, and excellent bending stability with the drain current remaining > 80% even after 500 bending. The detection limits of uric acid were in the range from 1 pM to mM. The MIP film was introduced to enhance the selectivity toward uric acid or adrenaline detection. 

### 4.2. Physical Sensors

A research team from the Research Institute for Intelligent Wearable Systems in Hong Kong [81] has presented exciting tactile sensors based on ultrathin all-solid organic electrochemical transistors (OECT). First, they introduced an innovative method for producing large-scale ultrafine polyaniline (PAni) fibers (UFPFs) using a modified wet-spinning technique. In contrast to conventional wet-spinning processes, they replaced less effective solvents with more suitable ones in the coagulation bath. This alteration reduced the viscosity of the gel fibers, enabling them to be stretched to an ultrafine morphology through an extremely high drawing ratio. Then, the team designed an ultrathin all-solid organic electrochemical transistor (OECT) leveraging the structural and electrochemical advantages of UFPFs. The OECT device operates with less than 1 V of driving voltage, significantly amplifies drain−source electrical signals while consuming minimal power, and effectively responds to both vertical pressure and horizontal friction forces at varying intensities.

**Figure 12:**
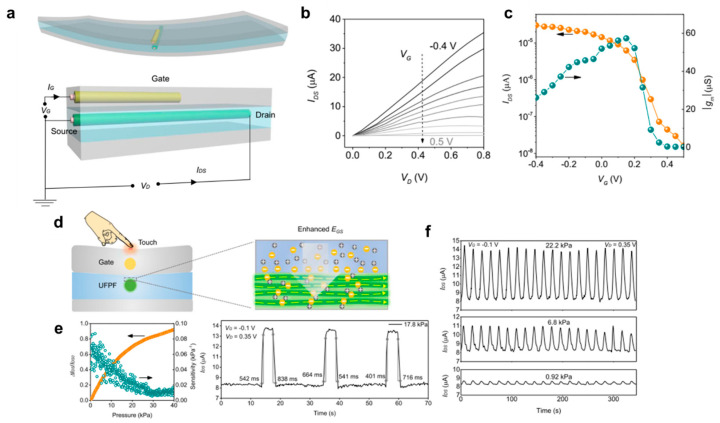
(**a**) Schematic diagram of the structure of an all-solid organic electrochemical transistor (OECT) consisting of three polymer layers, a silver wire serving as the gate electrode, and an ultra-flexible polymer film (UFPF) acting as the drain−source channel. (**b**) Output curve of the OECT, showing its operational performance. (**c**) The transfer curve of the OECT illustrates its behavior and characteristics. (**d**) Schematic diagram elucidating the underlying mechanism that accounts for the OECT’s response to external pressure. (**e**) Graphs presenting the relative change in drain−source current (ΔI_ds_/I_ds0_), sensitivity, and response time of the all-solid OECT when subjected to instantaneous pressure, including the application (rising edge) and removal (falling part) of pressure at 17.8 kPa. (**f**) Cyclic response exhibited by the OECT under three distinct pressure levels (0.92, 6.8, and 22.2 kPa), demonstrating its performance and reliability. Reproduced with permission from Fang et al. [81]. Copyright 2022, Springer Nature.

The formation of the tactile sensors based on OECTs is shown in Figure 12a. The OECT consists of three polymer layers. The upper layer serves as a dielectric coating and protective barrier, composed of cured polyurethane (PU), safeguarding the device from external influences [82]. A fibrous silver gate electrode, with a diameter of 7 μm, is embedded in the PU. The middle layer employs a PVA-H_3_PO_4_ gel to facilitate ion transport to and from the drain−source channel, with a UFPF immersed in the ion gel acting as the channel material. The bottom layer is also made of pure PU, providing structural support for the entire device. Despite having a long channel length (approximately 0.48 cm), considerably longer than conventional micrometer-scale devices, the all-solid OECT exhibits impressive amplification performance with a high on/off current ratio (>10^3^) at low voltages (<1 V), as shown in Figure 12b,c. They demonstrated that this all-solid OECT can effectively amplify small electrical signals in gel environments and respond to mechanical deformation as a tactile sensor. In Figure 12d, when vertical pressure is applied to the surface of the all-solid OECT, it adjusts the ion penetration due to improved gate-source electric field dynamics and the redistribution of intrinsic capacitance (Figure 12d right). With a V_G_ of −0.1 V and V_D_ of 0.35 V, there is a stable increase in drain−source current (I_DS_) with increasing pressure, achieving up to 92% amplification from 0 to 40 kPa, with a sensitivity level in the range of 0.01–0.1 kPa^−^^1^ (dark cyan dots in Figure 12e left). The right graph in Figure 12e illustrates the average rising and falling times under instantaneous pressure of 17.8 kPa, which are approximately 536 ms and 698 ms, respectively. In Figure 12f, these integrated parameters enable the all-solid OECT to effectively respond to different pressure levels ranging from 0.92 to 22.2 kPa. In addition to the response to the vertical pressure, the author demonstrated that the all-solid OECT responded to friction in the horizontal direction. The device stably responded to friction at different magnitudes from 1.84 to 5.55 kPa and different speeds from 4 to 20 mm/s during cyclic tests. 

**Table 1:** A summary of fiber-type transistor-based sensors is discussed in this review.

Transistor Type	Sensing Type	Configuration	Materials	Fiber Preparation Method	References
OECT	Glucose	Cross geometry	Polypyrrole/rGO/polyamide (PA) filament	In situ polymerization	[52]
OECT	Glucose	Cross geometry	PEDOT:PSS/Nylon fibers	Coating	[48]
OECT	Ions in human sweat	A single strand fiber	PEDOT:PSS fibers	Wet spinning	[28]
OECT	Saline concentration in human sweat	Two parallel fibers	PEDOT:PSS/cotton thread	Soaking	[41]
OECT	Ascorbic acid	Two parallel fibers	PEDOT:PSS fibers	Extrusion	[64]
OECT	Dopamine	Cross geometry	PPy/NFs/PA6 fiber	In situ polymerization	[34]
OECT	Uric acid	Two parallel fibers	PEDOT/rGO/cotton fiber	Reversed microemulsion polymerization	[76]
OECT	Tactile	Two parallel fibers	Polyaniline (PANi) fibers	Wet spinning	[81]

## 5. Conclusions and Future Perspectives

In this review, we have summarized recent developments in fiber-type transistors utilizing conjugated polymers and their applications in sensors, particularly chemical sensors including glucose, ionic concentration in human sweat, ascorbic acid, dopamine, uric acid sensors, and physical sensors such as tactile sensors. Despite notable progress, several challenges persist in the development and application of fiber-type transistor-based sensors, primarily related to stability, reproducibility, and selectivity in real-world environments. Furthermore, there is considerable potential for expanding the range of fiber-type electronic components and integrating them into textiles to enhance their functionality. For instance, weaving fiber-type sweat sensors together with tactile sensors and gas sensors into the fabric can enable the monitoring of the user’s health and surrounding environment. We anticipate that our manuscript will serve as a valuable resource for the development of innovative fiber-type sensors utilizing conjugated polymers. 

## Data Availability

The data presented in this study are available on request from the corresponding author.
